# Predictive biomarker of mortality in children with infectious diseases: a nationwide data analysis

**DOI:** 10.3389/fped.2024.1381310

**Published:** 2024-07-02

**Authors:** Shinya Miura, Tomohiro Katsuta, Yukitsugu Nakamura

**Affiliations:** ^1^ Department of Pediatrics, St. Marianna University School of Medicine, Kawasaki, Japan; ^2^ Graduate School of Public Health, Teikyo University, Tokyo, Japan

**Keywords:** biomarker, predict, infection, sepsis, child, mortality

## Abstract

Biomarkers play a crucial role in the early identification of high-risk children with infectious diseases. Despite their importance, few studies evaluated biomarkers' capabilities in predicting mortality. The aim of this study was to evaluate the biomarkers' predictive capabilities for mortality in children with infectious diseases. From an inpatient database covering ≥200 acute-care hospitals in Japan, we included children who underwent blood culture, and received antimicrobial treatment between 2012 and 2021. Biomarkers' results from the day of the initial blood culture were used. Biomarker discriminative capabilities were assessed using the area under receiver operating characteristic curves (AUCs). Of 11,365 eligible children with presumed infection, 1,378 (12.1%) required mechanical ventilation or vasoactive agents within 2 days of blood culture, and 100 (0.9%) died during admission. Of all children, 10,348 (91.1%) had community-onset infections and 1,017 (8.9%) had hospital-onset infections. C-reactive protein and white blood cell demonstrated limited discriminatory capabilities with AUCs of 0.44 [95% confidence interval (CI): 0.38–0.51] and 0.45 (95% CI: 0.39–0.52). In contrast, pH, prothrombin time-international normalized ratio, and procalcitonin exhibited strong discriminatory capabilities with AUCs of 0.77 (95% CI: 0.65–0.90), 0.77 (95% CI: 0.70–0.84) and 0.76 (95% CI: 0.29–1.00). In conclusions, our real-world data analysis suggested that C-reactive protein and white blood cell may not be reliable indicators for predicting mortality in children with presumed infection. These findings could warrant future studies exploring promising biomarkers, including those from blood gas analyses, coagulation studies and procalcitonin.

## 1 Introduction

Early identification and prompt treatment of children at elevated risk of mortality from infectious diseases are imperative, especially considering the significant number of pediatric deaths associated with serious infections (1). Several approaches have been proposed, including the use of predictive biomarkers for serious bacterial infections and the definition and identification of pediatric sepsis (2–4). However, the clinical application of these approaches remains challenging (5, 6).

Many of the existing studies evaluating the capabilities of biomarkers have focused primarily on serious bacterial infections as the outcome. Such outcome often has a low positive predictive value for mortality, casting doubts on the robustness of these biomarkers in predicting mortality (7–9). Moreover, although a few studies have evaluated biomarkers' capabilities to predict mortality, they have mostly been conducted in pediatric intensive care units (PICUs), which inherently admit children already deemed at risk of mortality. Consequently, this bias leaves an unaddressed knowledge gap regarding the risk stratification of children presenting with infectious diseases in emergency departments or general wards, where serious infections are often first recognized (4, 7, 10). Therefore, our study aimed to evaluate the predictive capabilities of biomarkers, tested when serious infections were initially suspected, for mortality in children admitted with infectious diseases across diverse settings in over 200 acute-care hospitals.

## 2 Method

### 2.1 Study design and participants

We performed a retrospective cohort study using an in/outpatient database managed by the Health, Clinic, and Education Information Evaluation Institute (Kyoto, Japan), which is a non-profit research service foundation, with support from Real World Data Co, Ltd (Kyoto, Japan). Details of the database are described elsewhere (11); briefly, it includes medical records, blood test results, and administrative claims data from over 200 hospitals from Kyushu to Hokkaido region of Japan. The database contains the following data: patient characteristics, diagnosis, medications and procedures during admission, and discharge status. This study was approved by the Institutional Review Board of Teikyo University (approval number: 22-019; May 20, 2020), and the procedures were in accordance with the ethical standards of the responsible committee on human experimentation and with the Helsinki Declaration of 1975. Due to the anonymity of the data the requirement of informed consent was waived.

We applied the following inclusion and exclusion criteria. We included children under 18 years of age who were admitted between January 2012 and December 2021, underwent blood culture, and received intravenous antimicrobial agents (antibiotics, antivirus and antifungals) starting within a window of 2 days before and after blood culture. Exclusion criteria were as follows: subsequent infectious events in previously included cases; neonates hospitalized since birth; use of mechanical ventilation or vasoactive agents priori to blood culture.

We stratified the children into the four severity groups: (i) short-term antimicrobial use for <4 days, (ii) antimicrobial treatment for four or more consecutive days, (iii) critical illness, defined as the initiation of mechanical ventilation or vasoactive agents within 2 days after blood culture, (iv) in-hospital death. Vasoactive agents included dopamine, epinephrine, norepinephrine, phenylephrine, or vasopressin.

### 2.2 Outcomes, biomarkers, and statistical analyses

The primary outcome was in-hospital mortality. We examined the following biomarkers: C-reactive protein (CRP), procalcitonin, white blood cell, platelet, prothrombin time international normalized ratio (PT-INR), pH, base excess, lactate, aspartate aminotransferase, alanine aminotransferase, bilirubin, creatine. The primary test was to calculate the area under a receiver-operator characteristic curve (AUC) of the results of biomarkers tested on the same day as blood culture for predicting in-hospital mortality. For each biomarker analysis, patients with missing data were excluded and no imputation for missing data was performed. We performed sensitivity analyses to assess the robustness of the primary test, by (i) using the worst values of biomarkers within 3 days and 7 days of blood culture, (ii) by time of infection onset, (iii) by dispositions, (iv) by age categories, (v) by excluding immunocompromised children, defined as those with malignant, haematological or immunological diagnoses, and (vi) by using an area under the precision-recall curve. Patients were classified as “community-onset infection” if neither blood culture nor antimicrobial initiation occurred on the first or second day of admission, or both, or “hospital-onset infection” if both blood culture and antimicrobial initiation occurred on the third day of admission or later (12). Patients were classified as “ICU” if admitted to an intensive care unit (ICU) or “ward” if admitted to a non-ICU setting on the day of blood culture collection. All analyses were performed using STATA 17 (StataCorp LLC, College Station, TX, USA).

## 3 Results

### 3.1 Patient characteristics

**Figure 1:**
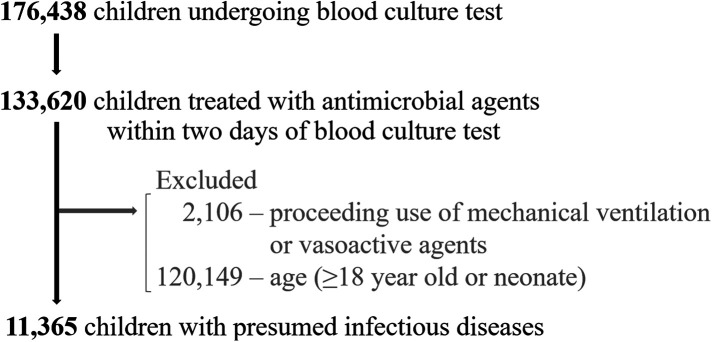
Patient flow.

Of 11,365 eligible children with presumed infectious diseases, 1,378 (12.1%) required mechanical ventilation or vasoactive agents within 2 days of blood culture, and 100 (0.9%) died during admission (Figure 1). The median age was 1 year (interquartile range: 0–5 years) with 4,958 (43.6%) being female. Of all children, 10,348 (91.1%) had community-onset infections and 1,017 (8.9%) had hospital-onset infections. By disposition, 2,024 (17.8%) children were admitted to ICUs on the day of blood culture (Table 1). Six biomarkers were tested in at least 80% of these children: CRP, white blood cell, platelet, aspartate aminotransferase, alanine aminotransferase, creatine. Meanwhile, pH, base excess and lactate were tested in 31.9%, 32.0% and 27.8%. PT-INR and Procalcitonin were tested in 24.1% and 16.6%, respectively (Table 2).

**Table 1:** Characteristics, therapies and outcomes of 11,365 children with presumed infectious diseases.

	*n*	(%)
Age, year, median (IQR)	1	(0–5)
Neonate	1,324	(11.6)
<1 year	2,978	(26.2)
2–5 years	4,462	(39.3)
6–18 years	2,601	(22.9)
Female	4,958	(43.6)
Complex chronic conditions		
Any	710	(6.2)
Cardiovascular	148	(1.3)
Neuromuscular	135	(1.2)
Malignancy	106	(0.9)
Hematological/immunological	105	(0.9)
Renal	92	(0.8)
Respiratory	83	(0.7)
Congenital/genetic	36	(0.3)
Transported from other hospitals	608	(5.3)
Community-onset infection^a^	10,348	(91.1)
Hospital-onset infection^b^	1,017	(8.9)
Therapies within 2 days of blood culture day		
Invasive ventilation	1,326	(11.7)
Vasoactive drugs	611	(5.4)
Renal replacement therapy	20	(0.2)
ECMO	21	(0.2)
Mortality	100	(0.9)

IQR, interquartile range; ICU, intensive care unit; ECMO, extracorporeal membrane oxygenation.

The number and percentage of variables were described when not specified.

^a^ Infection on admission was defined if neither blood culture nor antimicrobial initiation occurred on the first or second day of admission, or both.

^b^ Hospital-onset infection was defined if both blood culture and antimicrobial initiation occurred on the third day of admission or later.

**Table 2:** Biomarkers’ discriminatory capabilities for mortality—primary and sensitivity analyses.

	Primary test			Within 3 days			Within 7 days		
	*n*	AUC	(95% CI)	*n*	AUC	(95% CI)	*n*	AUC	(95% CI)
CRP	9,624	0.44	(0.38–0.51)	9,982	0.57	(0.51–0.64)	10,054	0.63	(0.45–1.00)
White blood cell	9,777	0.45	(0.39–0.52)	10,039	0.49	(0.37–0.61)	10,105	0.50	(0.56–0.69)
Procalcitonin	1,892	0.76	(0.29–1.00)	2,100	0.73	(0.42–0.55)	2,174	0.73	(0.44–0.56)
Platelet	9,801	0.68	(0.61–0.74)	10,070	0.72	(0.65–0.78)	10,132	0.75	(0.69–0.81)
PT-INR	2,735	0.77	(0.70–0.84)	3,156	0.87	(0.82–0.92)	3,325	0.86	(0.81–0.81)
pH	3,626	0.77	(0.65–0.90)	3,759	0.71	(0.59–0.84)	3,849	0.73	(0.61–0.85)
Base excess	3,638	0.62	(0.42–0.82)	3,776	0.65	(0.29–0.93)	3,865	0.64	(0.49–0.80)
Lactate	3,162	0.68	(0.52–0.84)	3,269	0.72	(0.49–0.82)	3,340	0.71	(0.57–0.85)
AST	9,661	0.72	(0.65–0.78)	9,960	0.75	(0.58–0.87)	10,067	0.76	(0.70–0.82)
ALT	9,634	0.69	(0.62–0.76)	9,940	0.72	(0.65–0.79)	10,049	0.74	(0.67–0.80)
Bilirubin	8,748	0.50	(0.42–0.57)	9,158	0.56	(0.50–0.63)	9,334	0.61	(0.55–0.67)
Creatine	9,626	0.69	(0.62–0.75)	9,935	0.72	(0.66–0.78)	10,042	0.74	(0.64–0.80)

In the primary test, the results of the biomarkers tested on the same day as the first blood culture were analyzed to evaluate the discriminatory capability of the biomarkers. As sensitivity analyses, the worst values of each biomarker within 3 and 7 days of blood culture were analyzed.

AUC, area under the receiver operating characteristic curve; CRP, C-reactive protein; PT-INR, prothrombin time international normalized ratio; AST, aspartate aminotransferase; ALT, alanine aminotransferase.

### 3.2 Predicting mortality

CRP and white blood cell showed poor discriminatory capabilities with AUCs of 0.44 [95% confidence interval (CI): 0.38–0.51] and 0.45 (95% CI: 0.39–0.52). On the other hand, pH, PT-INR and procalcitonin demonstrated strong discriminatory capabilities with AUCs of 0.77 (95% CI: 0.65–0.90), 0.77 (95% CI: 0.70–0.84) and 0.76 (95% CI: 0.29–1.00), respectively (Table 2 and Supplementary Figure S1).

### 3.3 Sensitivity analyses

Sensitivity analyses by using the worst values of the biomarkers within 3 and 7 days showed similar results. The AUCs of CRP increased slightly to 0.57 (95% CI: 0.51–0.64) and 0.63 (95% CI: 0.45–1.00), while the AUCs of white blood cell experienced negligible increases, standing at 0.49 (95% CI: 0.37–0.61) and 0.50 (95% CI: 0.56–0.69) for 3 and 7 days, respectively. In contrast, pH, PT-INR and procalcitonin consistently demonstrated strong discriminatory capabilities, irrespective of the duration of biomarkers’ tests (Table 2).

Sensitivity analyses by time of infection onset and disposition showed similar results (Supplementary Tables S1 and S2). CRP and white blood cell recorded AUCs ranging between 0.33–0.57 and 0.42–0.50, depending on the patient situation. Meanwhile, pH, PT-INR and procalcitonin exhibited AUCs spanning 0.65–0.87, 0.73–0.77 and 0.53–1.00, respectively. Sensitivity analyses by age and immunocompromised status showed similar results with the primary test (Supplementary Tables S3 and S4). Sensitivity analysis using precision-recall curves showed a 5-fold or greater increase in discriminatory accuracy for procalcitonin, PT-INR, pH, lactate, aspartate aminotransferase, alanine aminotransferase and creatine. In contrast, CRP and white blood cell did not improve the discriminatory accuracy (Supplementary Table S5).

### 3.4 Biomarkers by severity

**Figure 2:**
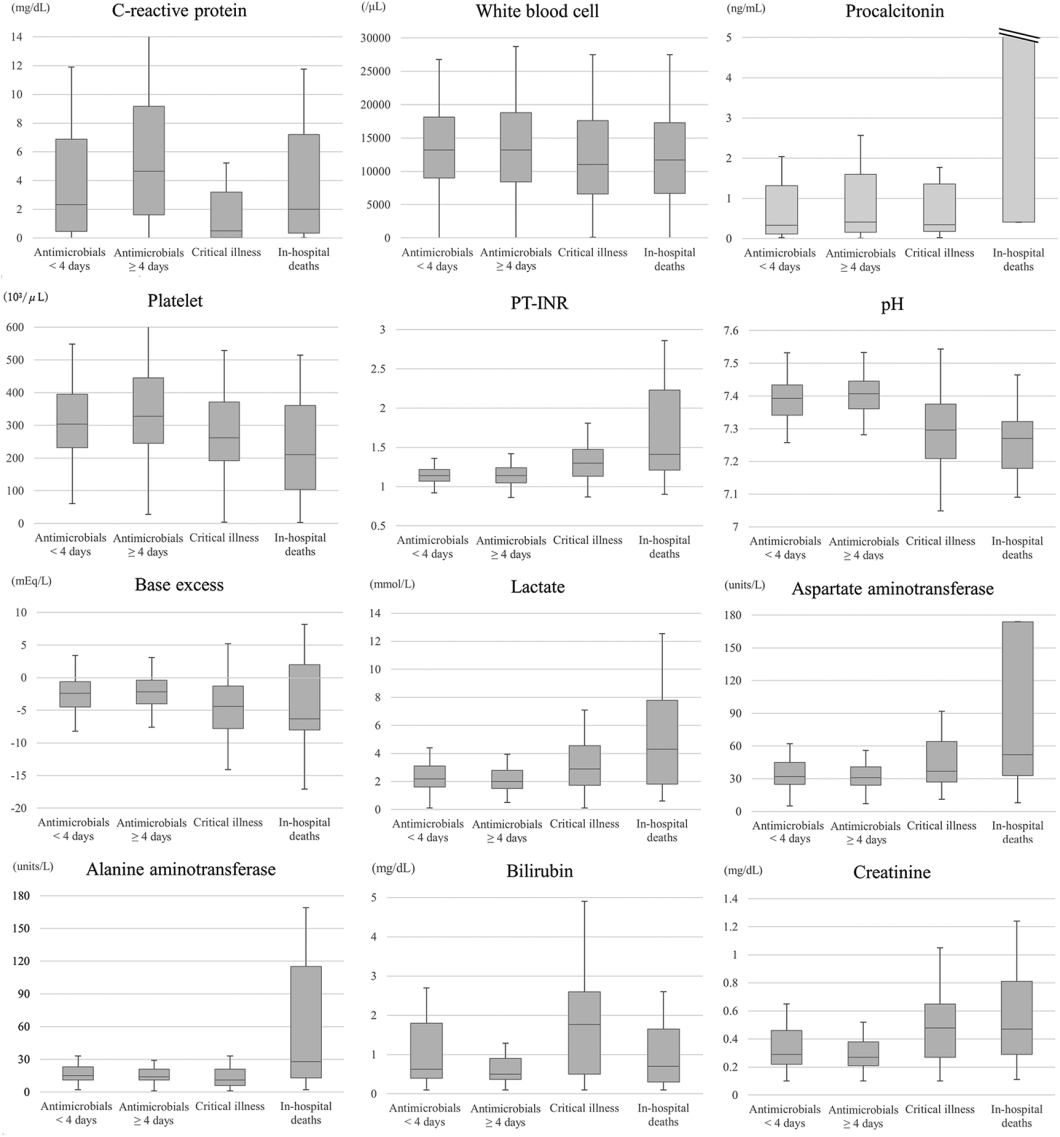
Biomarkers’ results by the severity of infection. Children were classified into the four severities groups: (i) short-term antimicrobial use for <4 days, (ii) antimicrobial treatment for ≥4 days, (iii) critical illness, defined as the initiation of mechanical ventilation or vasoactive agents within 2 days after blood culture, (iv) in-hospital death. PT-INR, prothrombin time international normalized ratio.

By severity groups, 2,807 (24.7%), 7,155 (63.0%), 1,303 (11.5%), and 100 (0.9%) children fell in (i) antimicrobials <4 days, (ii) antimicrobials ≥4 days, (iii) critical illness, and (iv) in-hospital death, respectively. In the higher severity groups, there were progressively worsening trends in eight biomarkers: procalcitonin, PT-INR, platelet, pH, lactate, base excess, aspartate aminotransferase, alanine aminotransferase (Figure 2). In contrast, CRP and white blood cell did not display consistent trends across varying severities.

## 4 Discussion

This analysis of real-world data evaluating biomarkers' capabilities to predict in-hospital mortality in children with presumed infectious diseases, demonstrated poor discriminatory capabilities in CRP and white blood cell and strong discriminatory capabilities in pH, PT-INR and procalcitonin.

While previous studies reported a strong discriminatory capability of CRP for serious bacterial infection, we found its poor discriminatory capability for mortality. One study reported the discriminatory capabilities of CRP with AUCs of 0.81 for serious bacterial infection and 0.43 for mortality in children presenting with suspected meningitis or pneumonia (13). One possible interpretation was that the outcome setting could significantly affect the biomarker's discrimination, given serious bacterial infections, defined as culture-proven invasive infections in many studies, do not necessarily indicate a high risk of death and need for intensive care (14). Another possible reason for our findings could be the delayed rise in CRP levels. A systematic review reported an improved discrimination of mortality with late-phase CRP levels compared to early-phase CRP levels recorded within 48 h. Similarly, our sensitivity analyses showed improved discrimination of CRP over extended observation periods. However, such marginal improvements did not suggest the clinical usefulness of CRP as an early predictor, as early recognition and timely treatment are crucial in managing severely-ill children (15). In addition, the cohort in our study may have included non-infectious cases characterized by high CRP levels and low mortality (e.g., Kawasaki disease, etc.). The international variation in disease incidence may have led to the discrepancy in CRP's performance. Furthermore, elevated CRP levels have been associated with improved outcomes in previous studies of selected cohorts with severe infections. This might be explained by spuriously low CRP levels due to an insufficient hepatic production, as CRP is a protein mainly synthesized in liver hepatocytes (16).

In our study, white blood cell count failed to discriminate the mortality. This aligned with previous studies showing its poor discrimination for mortality, with a limited sensitivity and specificity (8, 17).

Our study identified potentially predictive biomarkers including procalcitonin, pH, and PT-INR. Previous studies have shown that procalcitonin is useful in the early identification of children with serious bacterial infections at risk of mortality. It may be the time to discuss the implementation of procalcitonin testing among severely-ill children at risk of mortality, although the test should be judiciously reserved for properly-selected children due to its high cost. Similar to our findings, some of the biomarkers have been successfully used in ICU severity scores to accurately predict mortality e.g., PT-INR, aspartate aminotransferase, platelet, creatinine and pH in PELOD 2 and PRISM 3, although their validation in the general pediatric cohort hospitalized with infectious diseases is limited (17, 18). Other studies have demonstrated strong discriminatory capabilities of acidosis and PT-INR as a single biomarker or as a part of the prediction model (18–20). However, these studies have predominantly focused on specific conditions such as malaria or non-infectious cohorts (e.g., patients with traumatic injuries). Consequently, there is still a knowledge gap in the application of pH and PT-INR to predict mortality in children with infectious diseases. This gap warrants future studies to explore these potentially promising biomarkers.

### 4.1 Strengths and limitations

To the best of our knowledge, this was the first study to evaluate the biomarkers' capabilities to predict mortality in children in a huge sample size. However, this study had limitations. First, the inclusion criteria based on blood culture test and the initiation of intravenous antimicrobial agents may have resulted in different cohorts from those in other studies. However, we believe that our approach was the best available and reproducible to incorporate children with presumed infectious diseases into this analysis. Secondly, since not all children underwent every type of blood test, we could not directly compare the discriminatory capabilities of biomarkers. Third, in immunocompromised children, the discriminatory capabilities of biomarkers may have been affected due to their decreased baseline levels and suppressed biomarker responses. However, a sensitivity analysis by excluding immunocompromised children showed a similar result (Supplementary Table S2). Lastly, biomarkers that were tested less frequently may have been indicative of severely-ill children, who physicians thought needed more extensive evaluation. This may have influenced the calculated AUCs.

### 4.2 Conclusion

Our real-world data analysis suggested that CRP and white blood cell may not be reliable indicators for predicting mortality in children with presumed infectious diseases. These findings could warrant future studies exploring promising biomarkers, including those from blood gas analyses, coagulation studies and procalcitonin.

## Data Availability

The data analyzed in this study was obtained from Real World Data Co, Ltd (Kyoto, Japan). Requests to access these datasets should be directed to https://rwdata.co.jp/contact/.
