# Supplementary material for: Predictive biomarker of mortality in children with infectious diseases: a nationwide data analysis
Source: Front Pediatr. 2024 Jul 2;12:1381310. doi: 10.3389/fped.2024.1381310 (PMC11250247; doi:10.3389/fped.2024.1381310)
Supplement: Supplementary file 1 [file Datasheet1.docx]

**Supplementary materials**

Suppl Table 1. Sensitivity analysis by time of infection onset

|  | Community-onset infection | | |  | Hospital-onset infection | | |
| --- | --- | --- | --- | --- | --- | --- | --- |
|  | n=10,348 | | |  | n=1,017 | | |
|  | n | AUC | (95%CI) |  | n | AUC | (95%CI) |
| CRP | 8,795 | 0.33 | (0.23–0.42) |  | 829 | 0.57 | (0.48–0.66) |
| White blood cell | 8,936 | 0.50 | (0.40–0.60) |  | 841 | 0.49 | (0.40–0.57) |
| Procalcitonin | 1,797 | 1.00 | – |  | 95 | 0.53 | – |
| Platelet | 8,958 | 0.67 | (0.57–0.78) |  | 843 | 0.61 | (0.52–0.69) |
| PT-INR | 2,460 | 0.77 | (0.65–0.88) |  | 275 | 0.73 | (0.64–0.81) |
| pH | 3,451 | 0.83 | (0.67–0.98) |  | 175 | 0.65 | (0.45–0.85) |
| Base Excess | 3,462 | 0.66 | (0.37–0.95) |  | 176 | 0.61 | (0.32–0.89) |
| Lactate | 3,016 | 0.74 | (0.53–0.95) |  | 146 | 0.62 | (0.37–0.88) |
| AST | 8,903 | 0.77 | (0.67–0.87) |  | 758 | 0.67 | (0.58–0.75) |
| ALT | 8,877 | 0.79 | (0.69–0.88) |  | 757 | 0.55 | (0.46–0.65) |
| Bilirubin | 8,061 | 0.45 | (0.35–0.56) |  | 687 | 0.54 | (0.45–0.64) |
| Creatine | 8,858 | 0.75 | (0.67–0.83) |  | 768 | 0.62 | (0.52–0.71) |

In this analysis, biomarker’s discriminatory capabilities for mortality were evaluated by disposition.

Suppl Table 2. Sensitivity analysis by disposition

|  | Ward | | |  | ICU | | |
| --- | --- | --- | --- | --- | --- | --- | --- |
|  | n=9,320 | | |  | n=2,045 | | |
|  | n | AUC | (95%CI) |  | n | AUC | (95%CI) |
| CRP | 7,876 | 0.48 | (0.39–0.58) |  | 1,748 | 0.57 | (0.49–0.65) |
| White blood cell | 7,922 | 0.42 | (0.33–0.51) |  | 1,855 | 0.49 | (0.40–0.58) |
| Procalcitonin | – | | |  | 149 | 0.77 | (0.30–1.00) |
| Platelet | 7,949 | 0.71 | (0.62–0.80) |  | 1,852 | 0.60 | (0.49–0.71) |
| PT-INR | 1,992 | 0.75 | (0.64–0.86) |  | 743 | 0.74 | (0.63–0.84) |
| pH | 2,902 | 0.67 | (0.48–0.86) |  | 724 | 0.87 | (0.78–0.95) |
| Base Excess | 2,922 | 0.41 | (0.14–0.68) |  | 716 | 0.88 | (0.81–0.94) |
| Lactate | 2,454 | 0.47 | (0.23–0.71) |  | 708 | 0.86 | (0.75–0.96) |
| AST | 7,888 | 0.67 | (0.58–0.77) |  | 1,773 | 0.71 | (0.61–0.81) |
| ALT | 7,884 | 0.62 | (0.51–0.72) |  | 1,750 | 0.78 | (0.69–0.87) |
| Bilirubin | 7,014 | 0.45 | (0.34–0.56) |  | 1,734 | 0.36 | (0.27–0.44) |
| Creatine | 7,852 | 0.62 | (0.52–0.72) |  | 1,774 | 0.61 | (0.51–0.71) |

In this analysis, biomarker’s discriminatory capabilities for mortality were evaluated by disposition.

We did not examine AUCs if no or only one patient died within the categories.

Suppl Table 3. Sensitivity analysis by age

|  | Neonate | | |  | < 1 year | | |  | 1–5 years | | |  | 6–18 years | | |
| --- | --- | --- | --- | --- | --- | --- | --- | --- | --- | --- | --- | --- | --- | --- | --- |
|  | n=1,324 | | |  | n=2,978 | | |  | n=4,462 | | |  | n=2,601 | | |
|  | n | AUC | (95%CI) |  | n | AUC | (95%CI) |  | n | AUC | (95%CI) |  | n | AUC | (95%CI) |
| CRP | 1,127 | 0.59 | (0.49–0.69) |  | 2,503 | 0.45 | (0.32–0.58) |  | 3,814 | 0.34 | (0.18–0.50) |  | 2,180 | 0.52 | (0.38–0.66) |
| White blood cell | 1,169 | 0.42 | (0.32–0.51) |  | 2,583 | 0.59 | (0.41–0.76) |  | 3,837 | 0.47 | (0.34–0.60) |  | 2,188 | 0.46 | (0.33–0.59) |
| Procalcitonin | 115 | – | – |  | 464 |  | – |  | 873 | – | – |  | 440 | – | – |
| Platelet | 1,168 | 0.64 | (0.53–0.75) |  | 2,582 | 0.78 | (0.51–0.94) |  | 3,838 | 0.62 | (0.47–0.77) |  | 2,213 | 0.63 | (0.48–0.77) |
| PT-INR | 352 | 0.72 | (0.61–0.83) |  | 538 | 0.76 | (0.59–0.93) |  | 877 | 0.69 | (0.53–0.86) |  | 968 | 0.82 | (0.68–0.96) |
| pH | 408 | 0.62 | (0.41–0.84) |  | 1,061 | 0.55 | (0.00–1.00) |  | 1,464 | 0.96 | (0.93–1.00) |  | 693 | 0.67 | (0.36–0.99) |
| Base Excess | 409 | 0.74 | (0.53–0.96) |  | 1,062 | – | – |  | 1,470 | 0.64 | (0.00–1.00) |  | 697 | 0.54 | (0.16–0.93) |
| Lactate | 381 | 0.58 | (0.33–0.83) |  | 960 | 0.51 | (0.00–1.00) |  | 1,212 | 0.82 | (0.50–1.00) |  | 609 | 0.66 | (0.39–0.92) |
| AST | 1,098 | 0.56 | (0.44–0.69) |  | 2,543 | 0.77 | (0.60–0.94) |  | 3,826 | 0.82 | (0.71–0.93) |  | 2,194 | 0.78 | (0.66–0.89) |
| ALT | 1,086 | 0.63 | (0.49–0.76) |  | 2,532 | 0.80 | (0.64–0.96) |  | 3,824 | 0.77 | (0.63–0.91) |  | 2,192 | 0.72 | (0.59–0.84) |
| Bilirubin | 1,070 | 0.32 | (0.20–0.43) |  | 2,344 | 0.18 | (0.06–0.30) |  | 3,382 | 0.40 | (0.25–0.55) |  | 1,952 | 0.59 | (0.43–0.75) |
| Creatine | 1,104 | 0.47 | (0.35–0.60) |  | 2,534 | 0.61 | (0.44–0.77) |  | 3,806 | 0.76 | (0.60–0.92) |  | 2,182 | 0.70 | (0.58–0.81) |

In this analysis, biomarker’s discriminatory capabilities for mortality were evaluated by age category.

We did not examine AUCs if no or only one patient died within the categories.

Suppl Table 4. Sensitivity analysis by immunocompromised status

|  | Excluding  immunocompromised children | | |
| --- | --- | --- | --- |
|  | n | AUC | (95%CI) |
| CRP | 9,479 | 0.43 | (0.37–0.50) |
| White blood cell | 9,633 | 0.45 | (0.38–0.52) |
| Procalcitonin | 1,869 | 0.76 | (0.29–1.00) |
| Platelet | 9,655 | 0.68 | (0.61–0.74) |
| PT-INR | 2,670 | 0.77 | (0.70–0.84) |
| pH | 3,603 | 0.77 | (0.65–0.90) |
| Base Excess | 3,614 | 0.62 | (0.42–0.82) |
| Lactate | 3,140 | 0.68 | (0.52–0.84) |
| AST | 9,516 | 0.71 | (0.64–0.78) |
| ALT | 9,489 | 0.70 | (0.63–0.77) |
| Bilirubin | 8,613 | 0.50 | (0.42–0.57) |
| Creatine | 9,482 | 0.69 | (0.62–0.75) |

In this analysis, 202 children with malignant, haematological or immunological diagnoses were excluded.

Suppl Table 5. Sensitivity analysis by the precision-recall curve

|  | Biomarker's AUPRC | Baseline AUPRC | Ratio of biomarker/baseline |
| --- | --- | --- | --- |
| CRP | 0.009 | 0.009 | 1.1 |
| White blood cell | 0.009 | 0.009 | 1.0 |
| Procalcitonin | 0.007 | 0.001 | 6.7 |
| Hemoglobin | 0.032 | 0.009 | 3.4 |
| Platelet | 0.034 | 0.009 | 3.7 |
| PT-INR | 0.149 | 0.024 | 6.3 |
| pH | 0.050 | 0.006 | 8.6 |
| Base Excess | 0.016 | 0.005 | 3.5 |
| Lactate | 0.063 | 0.007 | 9.4 |
| AST | 0.113 | 0.009 | 12.6 |
| ALT | 0.087 | 0.009 | 9.7 |
| Bilirubin | 0.010 | 0.010 | 1.1 |
| Creatine | 0.048 | 0.009 | 5.1 |

In this analysis, the precision-recall curve was used instead of the sensitivity-specificity curve to estimate the area under the curve. The ratio of an AUPRC for each biomarker to the “baseline” AUPRC—a value for the AUPRC when a test randomly generates results in a tested cohort based on the prevalence of the outcome (i.e., mortality)—was calculated to interpret biomarker’s capabilities. For example, a procalcitonin test could contribute to a 6.7-fold increase in predictive accuracy.

AUPRC, area under the precision-recall curve.

Suppl Figure. ROC curves for mortality


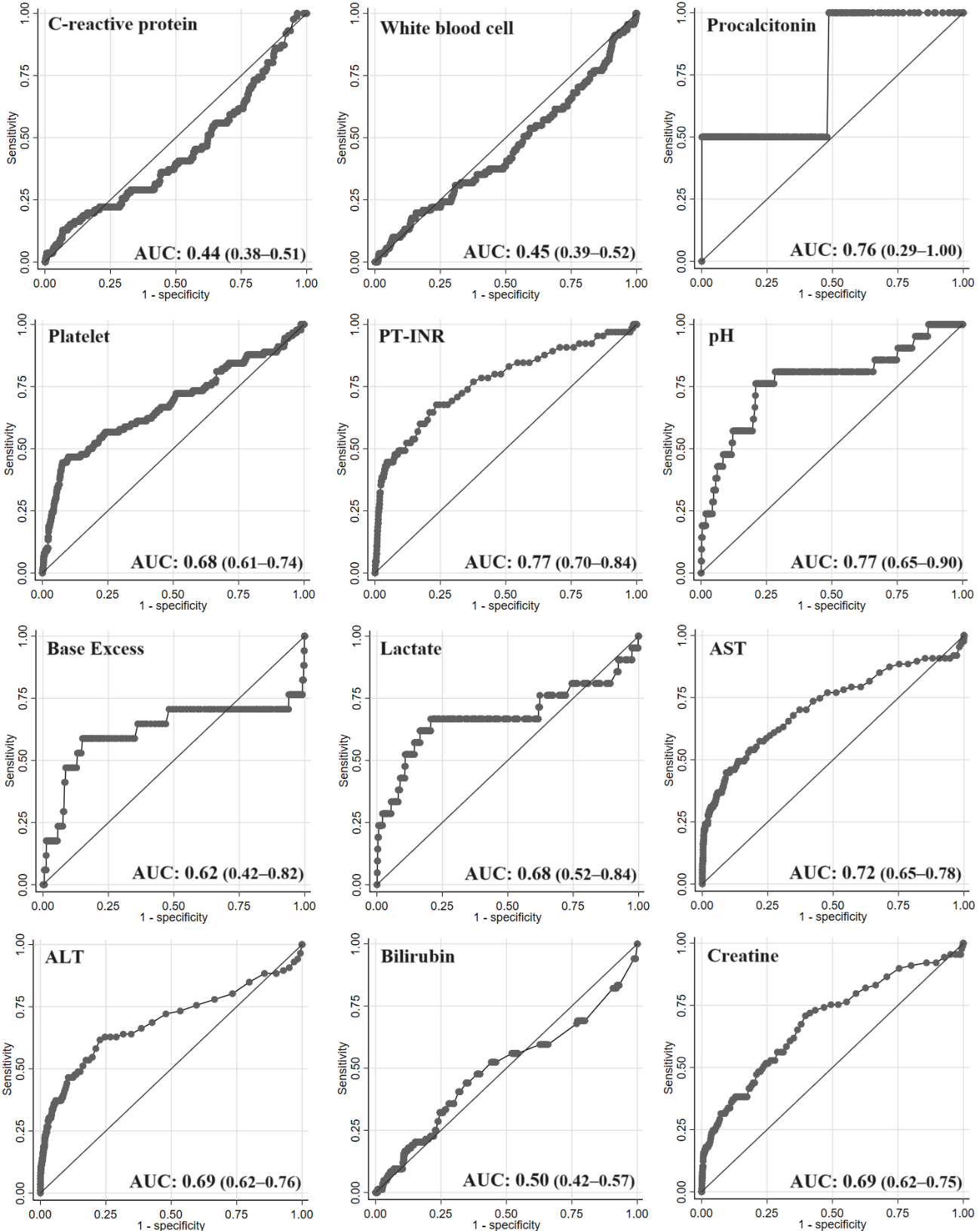


Negative values were used to predict mortality for platelets, pH and base excess.

AUC values with 95% confidence intervals were described for the biomarkers.

AUC, area under the receiver operating characteristic curve; PT-INR, prothrombin time international normalized ratio; AST, aspartate aminotransferase; ALT, alanine aminotransferase.
